# Supplementary material for: Genetic Architecture of Addiction-Relevant Behaviors in Outbred Sprague-Dawley Rats Reveals Loci for Anxiety-Like and Nociceptive Traits
Source: bioRxiv. 2026 Feb 19:2026.02.18.706481. Preprint. [Version 1] doi: 10.64898/2026.02.18.706481 (PMC12934780; doi:10.64898/2026.02.18.706481)

Table S1. Demographic Information. Behavioral assay, trait, sample size by sex, and testing age. Age values are mean ± SD in days at the time the assay was run. “M/F” gives the male and female counts. Within an assay block, all listed traits share the same sample size and age as the first row of that block unless otherwise noted. For trait definitions and procedures, see Methods Section 2.1.

Table S2. Covariates Regressed Out for Each Trait After the >2% Variance Screen. For each behavioral trait, the table lists covariates that individually explained more than 2% of the phenotypic variance and were therefore regressed out prior to downstream analyses. For each covariate, R^2 denotes the proportion of trait variance explained by that covariate alone, and Percent Variance Explained reports $100 \times R^2$. Sex met the >2% criterion for several EPM measures but not for locomotor or tail flick traits.

Table S3. Variant-Level Quality Control Summary. Filter Category indicates which variant-level quality control filter(s) each SNP failed: minor allele frequency (MAF < 0.05), Hardy–Weinberg equilibrium (HWE $P < 1 \times 10^{-7}$), and/or per-SNP genotype missingness (F-MISS above the specified thresholds). Rows labeled MAF only, HWE only, and F-MISS only correspond to SNPs that failed only that single filter, whereas combination rows correspond to SNPs that failed multiple filters. Total SNPs gives the total number of autosomal SNPs before QC. As noted in the main text, of the 9,088,280 SNPs with MAF < 0.05, 8,003,804 (88.1%) were monomorphic (MAF = 0).

Table S4. Summary Statistics for Behavioral Traits by Sex and Overall. For each trait within each behavioral assay (Locomotor Response to a Novel Environment, Elevated Plus Maze, Tail Flick), columns report Mean ± SD, Median, IQR, Min, and Max for Female, Male, and Overall. Values are computed from data before quantile normalization. SD, standard deviation; IQR, interquartile range.

Table S5. Phenotypic Correlations. Columns: Trait 1, Trait 2, rho, p_value, sig. rho is the Spearman rank correlation coefficient; p_value is two-sided; sig indicates significance at $p \leq 0.05$ (yes/no). Correlations were computed on covariate-adjusted, quantile-normalized residuals using pairwise complete observations. Trait definitions are in Methods Section 2.1.

Table S6.**SNP-Based Heritability of Behavioral Traits.** Heritability (h^2) was estimated with GCTA GREML using a genomic relationship matrix. The p value column reports the GCTA GREML likelihood-ratio test p-value for H_0: h = 0. Values that appeared as 0.000 in source files are shown as <0.001. Traits are ordered by h^2 from highest to lowest. Trait definitions are provided in Methods Section 2.1.

Table S7. Moderate-Impact Coding Variants within QTL Intervals. Summary of coding variants in linkage disequilibrium (LD) with trait lead SNPs. Coordinates are on the GRCr8 rat genome build. Candidate Gene is the gene harboring the coding variant and does not imply causality. QTL: peak marker of the QTL. SNP in LD with Peak Marker: moderate-impact missense variant (per SnpEff) in LD ($r^2 \geq 0.6$) with the peak marker. LD r^2 with Trait Top SNP:

pairwise LD r² between the listed variant and the trait lead SNP, estimated in the outbred Sprague-Dawley sample. SNP Change: HGVS c. annotation. Amino Acid Change: HGVS p. annotation.

Table S8. **Summary of QTLs.** Peak markers and linkage-disequilibrium (LD) intervals for quantitative trait loci. Coordinates are on the GRCr8 rat genome build. $-\log_{10}P$ is the test statistic; Significance_Level indicates the 5% or 10% genome-wide threshold derived by permutation. A1 is the effect allele, A2 is the other allele, and Freq is the effect-allele frequency. b is the per-allele effect on the quantile-normalized trait with standard error se. The LD interval is defined as all variants with $r^2 \geq 0.6$ around the peak, reported as Start and Stop base-pair positions and physical Size in megabases.

Table S9 Shared Phenotypes between the Outbred Sprague-Dawley (this study) and the bHR/bLR F_2 Rats (Chitre et al., 2023). This table shows the mean age ± SD at the time of behavioral testing for the rats in the two studies.

Table S10. Phenotypic Correlations (Spearman rho) between Elevated Plus Maze (EPM) Measures and Locomotor Traits in Outbred Sprague-Dawley (this study) and bHR/bLR F_2 rats (Chitre et al., 2023). This table reports Spearman rho and two-sided p for each overlapping EPM × Locomotor trait pair measured in both studies. Values are shown as: rho_Outbred, p_Outbred, rho_F2, p_F2. Only the traits present in both studies appear in the rows of this table. Outbred Sprague-Dawley refers to this study; bHR/bLR F_2 refers to the intercross reported in Chitre et al., 2023.

Table S11. bHR/bLR F_2 (RN6) QTLs lifted to GRCr8 and Queried in Outbred Sprague-Dawley. This table lists QTLs discovered in the bHR/bLR F_2 study on RN6 and their lifted coordinates on GRCr8 used to query the outbred Sprague-Dawley GWAS. Columns: Behavioral Test, Trait (F_2 Label); Original Top SNP: F_2 peak SNP formatted as chr:pos; Original Top SNP $-\log_{10}P$: F_2 peak strength; Original LD Start: F_2 LD window start in bp; Original LD Stop: F_2 LD window stop in bp; Original Interval Size: F_2 LD window size in Mb computed as (stop − start)/1e6; Queried Trait: queried outbred trait; Lifted Top SNP: lifted peak SNP on GRCr8 formatted as chr:pos, NA if absent; Lifted LD Start: lifted LD window start in bp on GRCr8; Lifted LD Stop: lifted LD window stop in bp on GRCr8; Lifted Interval Size: lifted window size in Mb; Best $-\log_{10}P$: best signal in outbred Sprague-Dawleys within the lifted window reported as $-\log_{10}P$; Best SNP: position of that best signal formatted as chr:pos. Sizes are in megabases and rounded.

Table S12. Outbred Sprague-Dawley (GRCr8) Loci Lifted to RN6 and Queried in bHR/bLR F_2. This table lists QTLs discovered in outbred Sprague-Dawleys on GRCr8 and their lifted coordinates on RN6 used to query the bHR/bLR F_2 GWAS. Columns: Behavioral Test, Trait: outbred trait label; Original Top SNP: outbred peak SNP formatted as chr:pos; Original Top SNP -log10P: outbred peak strength; Original LD Start: outbred LD window start in bp; Original LD Stop: outbred LD window stop in bp; Original Interval Size (Mb): outbred window size in Mb; Queried Trait: queried F_2 trait; Lifted Top SNP: lifted peak SNP on RN6 formatted as chr:pos,

NA if absent; Lifted LD Start: lifted LD window start in bp on RN6; Lifted LD Stop: lifted LD window stop in bp on RN6; Lifted Interval Size (MB): lifted window size in Mb; Best −log10P: best signal in F_2 within the lifted window reported as −log10P; best SNP: position of that best signal formatted as chr:pos Sizes are in megabases and rounded.

Table S1. Demographic Information.			
**Behavioral Assay**	**Trait**	**Sample Size (M/F)**	**Mean Age (Days) ± SD**
Locomotor Response to a Novel Environment	Lateral Locomotor Score	650 (327/323)	77.68 ± 0.90
	Total Locomotor Score		
	Rearing Locomotor Score		
Elevated Plus Maze	EPM Distance Traveled	654 (327/327)	85.22 ± 1.55
	EPM Open Arm Entries		
	EPM Percent Time in Open Arms		
	EPM Open Arm Time Ratio		
	EPM Open Arm Time		
	EPM Velocity		
	EPM Time Immobile		
Tail Flick	Tail Flick Latency	534 (267/267)	94.03 ± 3.48

Table S2. Covariates Regressed Out for Each Trait After the >2% Variance Screen.			
**Trait**	**Covariate Regressed Out**	**R2**	**Percent Variance Explained**
EPM Open Arm Time Ratio	Experimenter: EH_SK	0.074	7.4
EPM Open Arm Time Ratio	Sex: M	0.071	7.1
EPM Open Arm Time Ratio	Experimenter: EH_KA	0.02	2
EPM Open Arm Time	Experimenter: EH_SK	0.092	9.2
EPM Open Arm Time	Sex: M	0.065	6.5
EPM Open Arm Time	Experimenter: EH_JS	0.028	2.8
EPM Open Arm Time	Experimenter: EH_KA	0.027	2.7
EPM Open Arm Entries	Sex: M	0.053	5.3
EPM Open Arm Entries	Experimenter: EH_SM	0.033	3.3
EPM Open Arm Entries	room: R04 or R08	0.032	3.2
EPM Open Arm Entries	Experimenter: EH_JS	0.022	2.2
EPM Percent Time in Open Arms	Experimenter: EH_SK	0.092	9.2
EPM Percent Time in Open Arms	Sex: M	0.065	6.5
EPM Percent Time in Open Arms	Experimenter: EH_JS	0.028	2.8
EPM Percent Time in Open Arms	Experimenter: EH_KA	0.027	2.7
EPM Distance Traveled	Experimenter: EH_SM	0.04	4
EPM Distance Traveled	room: R04 or R08	0.038	3.8
EPM Distance Traveled	Sex: M	0.02	2
EPM Velocity	Experimenter: EH_SM	0.04	4
EPM Velocity	room: R04 or R08	0.038	3.8
EPM Velocity	Sex: M	0.02	2
EPM Time Immobile	Experimenter: EH_JS	0.031	3.1
EPM Time Immobile	Test Order Per Day: 29	0.02	2

Table S3. Variant-Level Quality Control Summary.

Filter Category	Count
MAF only	8,042,158
HWE only	1,460
F-MISS only	3,911,924
MAF+HWE	2,415
MAF+F-MISS	1,041,835
HWE+F-MISS	229,310
MAF+HWE+F-MISS	1,872
Total SNPs	14,874,570

Table S4. Summary Statistics for Behavioral Traits by Sex and Overall.

Behavioral Assay	Trait	Female					Male					Overall				
		Mean ± SD	Median	IQR	Min	Max	Mean ± SD	Median	IQR	Min	Max	Mean ± SD	Median	IQR	Min	Max
Locomotor Response to a Novel Environment	Total Locomotor Score	794.57 ± 259.14	746.00	369.50	301.00	1,577.00	527.89 ± 192.59	498.00	248.00	172.00	1,413.00	660.41 ± 264.11	608.00	350.50	172.00	1,577.00
	Lateral Locomotor Score	251.45 ± 81.86	239.00	107.00	58.00	550.00	181.91 ± 66.78	170.00	91.00	42.00	429.00	216.47 ± 82.31	204.00	108.75	42.00	550.00
	Rearing Locomotor Score	543.12 ± 189.13	510.00	264.00	172.00	1,119.00	345.98 ± 135.52	325.00	173.50	104.00	987.00	443.94 ± 191.58	412.00	245.75	104.00	1,119.00
Elevated Plus Maze	EPM Open Arm Time	61.30 ± 35.58	58.03	52.15	0.00	160.50	43.75 ± 36.59	39.30	52.62	0.00	160.26	52.53 ± 37.12	48.78	53.39	0.00	160.50
	EPM Open Arm Entries	8.10 ± 3.94	8.00	5.00	0.00	26.00	6.46 ± 4.15	6.00	6.00	0.00	24.00	7.28 ± 4.13	7.00	6.00	0.00	26.00
	EPM Percent Time in Open Arms	20.50 ± 11.90	19.41	17.44	0.00	53.68	14.63 ± 12.24	13.14	17.60	0.00	53.60	17.57 ± 12.41	16.32	17.86	0.00	53.68
	EPM Open Arm Time Ratio	27.20 ± 15.56	25.96	23.01	0.00	81.22	19.07 ± 15.81	17.18	23.29	0.00	66.09	23.13 ± 16.19	21.66	24.38	0.00	81.22
	EPM Distance Traveled	1,683.70 ± 353.29	1,690.33	454.93	522.42	2,620.64	1,592.21 ± 310.72	1,567.28	356.44	725.40	2,825.23	1,637.96 ± 335.57	1,621.08	428.46	522.42	2,825.23
	EPM Velocity	5.63 ± 1.18	5.66	1.52	1.75	8.77	5.33 ± 1.04	5.25	1.19	2.43	9.46	5.48 ± 1.12	5.42	1.44	1.75	9.46
	EPM Time Immobile	116.95 ± 23.78	114.70	25.32	70.50	246.83	110.65 ± 18.39	108.83	22.52	70.53	188.20	113.80 ± 21.47	111.50	24.61	70.50	246.83
Tail Flick	Tail Flick Latency	8.42 ± 4.24	8.08	6.56	0.52	15.00	8.24 ± 4.26	7.96	6.39	0.88	15.00	8.33 ± 4.24	8.00	6.40	0.52	15.00

Table S5. Phenotypic Correlations.

Trait 1	Trait 2	rho	p_value	sig
Lateral Locomotor Score	Total Locomotor Score	0.92	3.40E-265	yes
Rearing Locomotor Score	Total Locomotor Score	0.98	0.00E+00	yes
Rearing Locomotor Score	Lateral Locomotor Score	0.84	1.80E-174	yes
EPM Open Arm Time	Total Locomotor Score	0.12	1.47E-03	yes
EPM Open Arm Time	Lateral Locomotor Score	0.11	6.18E-03	yes
EPM Open Arm Time	Rearing Locomotor Score	0.13	1.05E-03	yes
EPM Open Arm Entries	Total Locomotor Score	0.11	5.50E-03	yes
EPM Open Arm Entries	Lateral Locomotor Score	0.1	8.96E-03	yes
EPM Open Arm Entries	Rearing Locomotor Score	0.11	5.37E-03	yes
EPM Open Arm Entries	EPM Open Arm Time	0.72	5.60E-105	yes
EPM Percent Time in Open Arms	Total Locomotor Score	0.12	1.47E-03	yes
EPM Percent Time in Open Arms	Lateral Locomotor Score	0.11	6.18E-03	yes
EPM Percent Time in Open Arms	Rearing Locomotor Score	0.13	1.05E-03	yes
EPM Percent Time in Open Arms	EPM Open Arm Time	1	0.00E+00	yes
EPM Percent Time in Open Arms	EPM Open Arm Entries	0.72	5.60E-105	yes
EPM Open Arm Time Ratio	Total Locomotor Score	0.1	9.08E-03	yes
EPM Open Arm Time Ratio	Lateral Locomotor Score	0.09	2.95E-02	yes
EPM Open Arm Time Ratio	Rearing Locomotor Score	0.11	6.45E-03	yes
EPM Open Arm Time Ratio	EPM Open Arm Time	0.96	0.00E+00	yes
EPM Open Arm Time Ratio	EPM Open Arm Entries	0.71	4.30E-103	yes
EPM Open Arm Time Ratio	EPM Percent Time in Open Arms	0.96	0.00E+00	yes
EPM Distance Traveled	Total Locomotor Score	0.11	3.68E-03	yes
EPM Distance Traveled	Lateral Locomotor Score	0.13	6.11E-04	yes
EPM Distance Traveled	Rearing Locomotor Score	0.1	8.61E-03	yes
EPM Distance Traveled	EPM Open Arm Time	0.41	7.00E-28	yes
EPM Distance Traveled	EPM Open Arm Entries	0.45	2.56E-33	yes
EPM Distance Traveled	EPM Percent Time in Open Arms	0.41	7.00E-28	yes
EPM Distance Traveled	EPM Open Arm Time Ratio	0.38	9.40E-24	yes
EPM Velocity	Total Locomotor Score	0.11	0.00373	yes

EPM Velocity	Lateral Locomotor Score	0.13	0.000632	yes
EPM Velocity	Rearing Locomotor Score	0.1	0.00867	yes
EPM Velocity	EPM Open Arm Time	0.41	6.38E-28	yes
EPM Velocity	EPM Open Arm Entries	0.45	2.46E-33	yes
EPM Velocity	EPM Percent Time in Open Arms	0.41	6.38E-28	yes
EPM Velocity	EPM Open Arm Time Ratio	0.38	8.53E-24	yes
EPM Velocity	EPM Distance Traveled	1	0	yes
EPM Time Immobile	Total Locomotor Score	-0.02	0.665	no
EPM Time Immobile	Lateral Locomotor Score	-0.04	0.278	no
EPM Time Immobile	Rearing Locomotor Score	-0.01	0.826	no
EPM Time Immobile	EPM Open Arm Time	-0.28	1.20E-13	yes
EPM Time Immobile	EPM Open Arm Entries	-0.35	2.06E-20	yes
EPM Time Immobile	EPM Percent Time in Open Arms	-0.28	1.20E-13	yes
EPM Time Immobile	EPM Open Arm Time Ratio	-0.25	4.47E-11	yes
EPM Time Immobile	EPM Distance Traveled	-0.84	4.00E-177	yes
EPM Time Immobile	EPM Velocity	-0.84	2.90E-177	yes
Tail Flick Latency	Total Locomotor Score	0.01	0.739	no
Tail Flick Latency	Lateral Locomotor Score	0.05	0.247	no
Tail Flick Latency	Rearing Locomotor Score	-0.01	0.907	no
Tail Flick Latency	EPM Open Arm Time	-0.06	0.188	no
Tail Flick Latency	EPM Open Arm Entries	-0.05	0.283	no
Tail Flick Latency	EPM Percent Time in Open Arms	-0.06	0.188	no
Tail Flick Latency	EPM Open Arm Time Ratio	-0.07	0.12	no
Tail Flick Latency	EPM Distance Traveled	-0.08	0.0742	no
Tail Flick Latency	EPM Velocity	-0.08	0.0727	no
Tail Flick Latency	EPM Time Immobile	0.14	0.00128	yes

Table S6.SNP-Based Heritability of Behavioral Traits.

Trait	SNP h2 ± SE	P value
Lateral Locomotor Score	0.375 ± 0.083	<0.001
Total Locomotor Score	0.368 ± 0.086	<0.001
Rearing Locomotor Score	0.351 ± 0.087	<0.001
EPM Velocity	0.298 ± 0.086	<0.001
EPM Distance Traveled	0.296 ± 0.086	<0.001
EPM Time Immobile	0.276 ± 0.084	<0.001
EPM Open Arm Time Ratio	0.156 ± 0.082	0.028
Tail Flick Latency	0.153 ± 0.094	0.033
EPM Percent Time in Open Arms	0.143 ± 0.083	0.054
EPM Open Arm Time	0.143 ± 0.083	0.054
EPM Open Arm Entries	0.136 ± 0.084	0.061

Table S7. Moderate-Impact Coding Variants within QTL Intervals.

Trait	QTL	Candidate Gene	SNP in LD with Peak Marker	LD r^2 with Trait Top SNP	SNP Change	Amino Acid Change
EPM Percent Time in Open Arms	Chr1:267.773 Mb	*Eno4*	Chr1:268,084,478	0.681	c.187G>T	p.Ala63Ser
EPM Open Arm Time Ratio	Chr1:268.059 Mb	*Eno4*	Chr1:268,084,478	0.691	c.187G>T	p.Ala63Ser
EPM Open Arm Time	Chr1:267.773 Mb	*Eno4*	Chr1:268,084,478	0.681	c.187G>T	p.Ala63Ser
Tail Flick Latency	Chr17:30.015 Mb	*Tex56p*	Chr17:30,152,157	0.839	c.552C>G	p.Asp184Glu

Table S8. Summary of QTLs.

	Peak Marker										LD Interval		
Trait	Chr	SNP	Position (bp)	-log10P	Significance_Level	A1	A2	Freq	b	se	Start (bp)	Stop (bp)	Size (Mb)
EPM Percent Time in Open Arms	Chr1	Chr1:267.773 Mb	267,772,657	7.283	5%	A	T	0.453	-0.314	0.058	267,526,572	268,502,952	0.55
EPM Open Arm Time		Chr1:267.773 Mb	267,772,657	7.283	5%	A	T	0.453	-0.314	0.058	267,526,572	268,502,952	0.55
EPM Open Arm Time Ratio		Chr1:268.059 Mb	268,059,169	6.735	5%	G	A	0.455	-0.303	0.058	267,527,231	268,486,673	0.55
EPM Time Immobile	Chr14	Chr14:102.124 Mb	102,123,644	5.838	10%	T	C	0.498	0.272	0.057	101,958,495	102,170,686	0.13
Tail Flick Latency	Chr17	Chr17:30.015 Mb	30,015,342	6.267	5%	A	T	0.089	-0.546	0.109	29,944,792	30,363,871	0.41

Table S9. Shared Phenotypes between the Outbred Sprague-Dawley (this study) and the bHR/bLR F_2 Rats (Chitre et al., 2023).

Assay	Shared Phenotype	Outbred Age (days; mean ± SD)	F2 Age (days; mean ± SD)	Δ Age (weeks)
Locomotor response to novelty	Lateral Locomotor Score	77.7 ± 0.9	123 ± 5	~6.5
	Rearing Locomotor Score			
	Total Locomotor Score			
Elevated plus maze	EPM Distance Traveled	85.2 ± 1.6	123 ± 5	~5.4
	EPM Percent Time in Open Arms			
	EPM Time Immobile			

Table S10. Phenotypic Correlations (Spearman rho) between Elevated Plus Maze (EPM) Measures and Locomotor Traits in Outbred Sprague-Dawley (this study) and bHR/bLR F_2 rats (Chitre et al., 2023).

EPM Measure	Locomotor Measure	rho_Outbred	p_Outbred	rho_F2	p_F2
EPM Distance Traveled	Lateral Locomotor Score	0.13	1.00E-03	0.402	0.00E+00
EPM Distance Traveled	Total Locomotor Score	0.11	4.00E-03	0.424	0.00E+00
EPM Distance Traveled	Rearing Locomotor Score	0.1	9.00E-03	0.414	0.00E+00
EPM Percent Time in Open Arms	Lateral Locomotor Score	0.11	6.00E-03	0.244	5.39E-07
EPM Percent Time in Open Arms	Total Locomotor Score	0.12	1.00E-03	0.262	7.22E-08
EPM Percent Time in Open Arms	Rearing Locomotor Score	0.13	1.00E-03	0.255	1.70E-07
EPM Time Immobile	Lateral Locomotor Score	-0.04	2.78E-01	-0.358	7.80E-14
EPM Time Immobile	Total Locomotor Score	-0.02	6.65E-01	-0.417	0.00E+00
EPM Time Immobile	Rearing Locomotor Score	-0.01	8.26E-01	-0.432	0.00E+00

Table S11. bHR/bLR F_2 (RN6) QTLs lifted to GRCr8 and Queried in Outbred Sprague-Dawley.

Study & Trait		Origin (bHR/bLR F2, RN6)					Lifted & Queried (Outbred Sprague-Dawley, GRCr8)						
Behavioral Test	Trait (F2 Label)	Original Top SNP	Original Top SNP -log10P	Original LD Start	Original LD Stop	Original Interval Size (Mb)	Queried Trait	Lifted Top SNP	Lifted LD Start	Lifted LD Stop	Lifted Interval Size (Mb)	Best −log10P	Best SNP
Locomotor Response to a Novel Environment	Total Locomotor Score	Chr1:95,096,740	7.66	94,322,277	95,263,107	0.941	Total Locomotor Score	NA	99,762,095	100,692,809	0.931	1.61	Chr1:99,782,419
	Lateral Locomotor Score	Chr1:95,096,740	7.00	94,322,277	95,263,107	0.941	Lateral Locomotor Score	NA	99,762,095	100,692,809	0.931	1.30	Chr1:99,782,419
	Rearing Locomotor Score	Chr1:95,087,956	6.89	94,322,277	95,263,107	0.941	Rearing Locomotor Score	NA	99,762,095	100,692,809	0.931	1.66	Chr1:99,862,586
	Total Locomotor Score	Chr1:107,298,166	11.88	107,126,050	107,363,578	0.238	Total Locomotor Score	NA	110,477,039	110,718,512	0.241	1.45	Chr1:110,485,783
	Lateral Locomotor Score	Chr1:107,181,408	12.09	107,126,050	107,451,256	0.325	Lateral Locomotor Score	NA	110,477,039	110,806,642	0.33	1.12	Chr1:110,485,783
	Rearing Locomotor Score	Chr1:107,298,166	10.97	107,126,050	107,363,578	0.238	Rearing Locomotor Score	NA	110,477,039	110,718,512	0.241	1.42	Chr1:110,485,783
	Lateral Locomotor Score	Chr7:84,269,306	7.91	83,274,530	90,251,798	6.977	Lateral Locomotor Score	NA	77,406,383	83,965,203	6.559	2.26	Chr7:80,705,907
EPM	EPM Distance Traveled	Chr1:94,914,942	7.13	94,107,350	95,263,107	1.156	EPM Distance Traveled	NA	99,551,713	100,692,809	1.141	0.47	Chr1:99,888,981

Table S12. Outbred Sprague-Dawley (GRCr8) Loci Lifted to RN6 and Queried in bHR/bLR F_2.

Study & Trait		Origin (Outbred Sprague-Dawley, GRCr8)					Lifted & Queried (bHR/bLR F2, RN6)						
**Behavioral Test**	**Trait**	**Original Top SNP**	**Original Top SNP -log10P**	**Original LD Start**	**Original LD Stop**	**Original Interval Size (Mb)**	**Queried Trait**	**Lifted Top SNP**	**Lifted LD Start**	**Lifted LD Stop**	**Lifted Interval Size (MB)**	**Best −log10P**	**Best SNP**
EPM	Percent Time in Open Arms	Chr1:267,772,657	7.28	267,526,572	268,502,952	0.55	EPM Percent Time in Open Arms	NA	279,542,985	280,525,689	0.98	2.23	Chr1:280,052,026
	Time Immobile	Chr14:102,123,644	5.84	101,958,495	102,170,686	0.13	EPM Time Immobile	NA	108,554,098	108,766,958	0.21	1.62	Chr14:108,679,940

Supplementary Figure S1. Manhattan plots for each behavioral trait. SNPs are plotted by genomic position on GRCr8 (x) and −log10P from LOCO mixed-model GWAS (y). Horizontal lines show genome-wide (5% at −log10P = 5.98) and suggestive (10% at −log10P = 5.60) thresholds derived by permutation.

EPM Distance Traveled

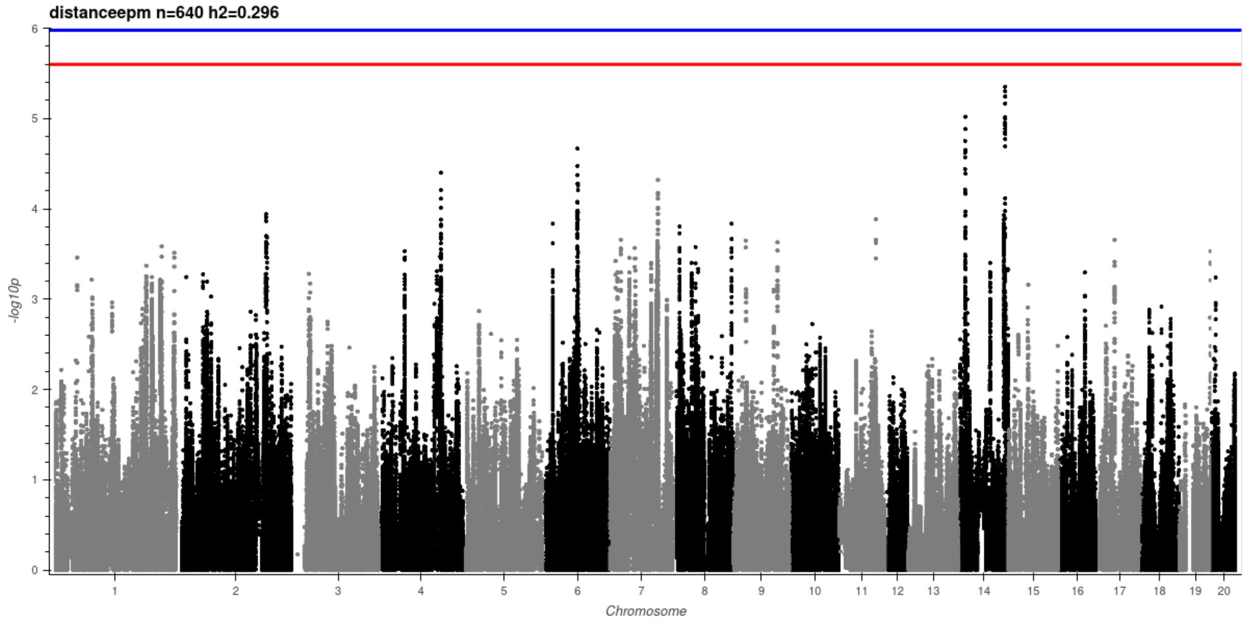

EPM Open Arm Entries

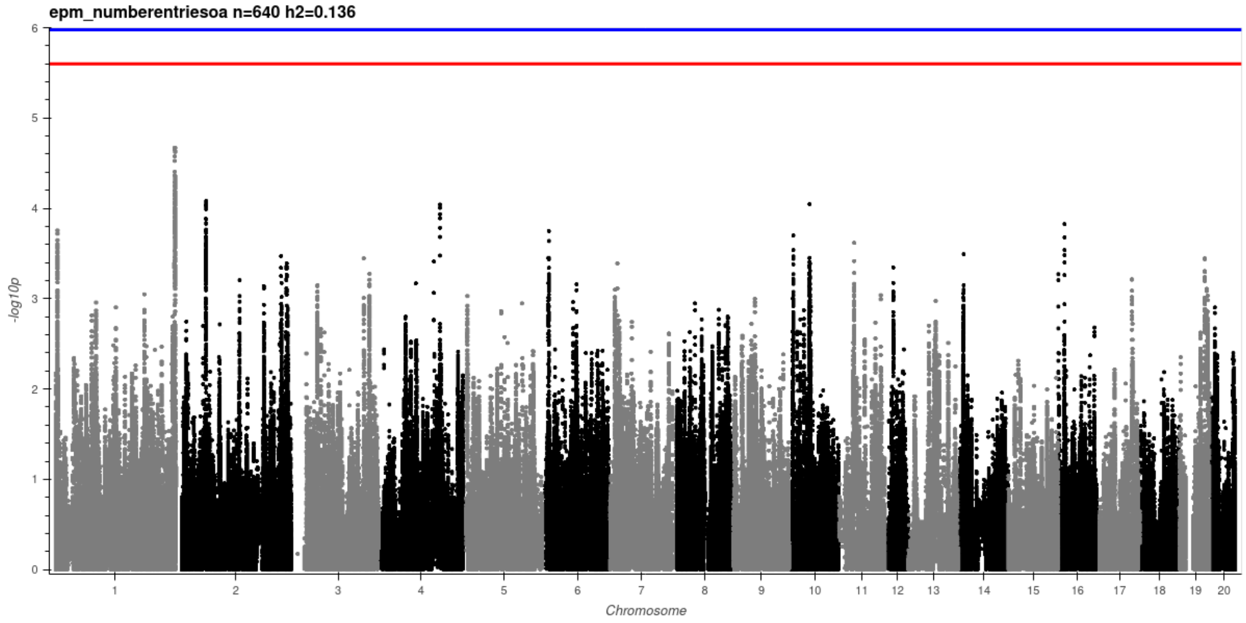

EPM Percent Time in Open Arms

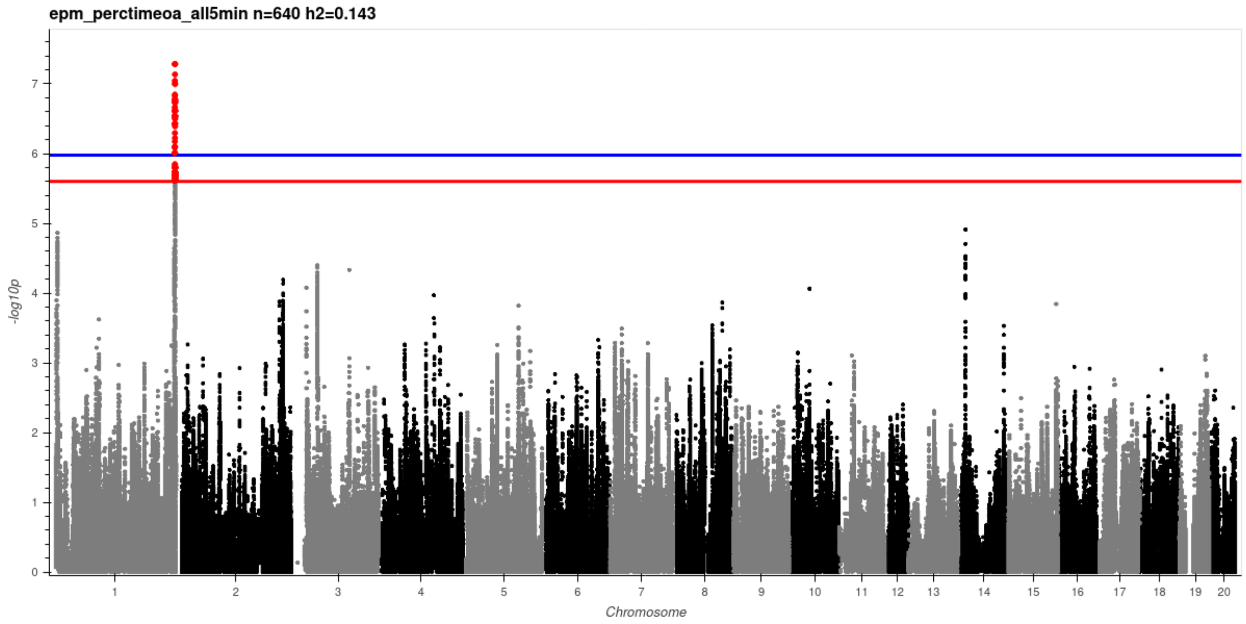

EPM Open Arm Time Ratio

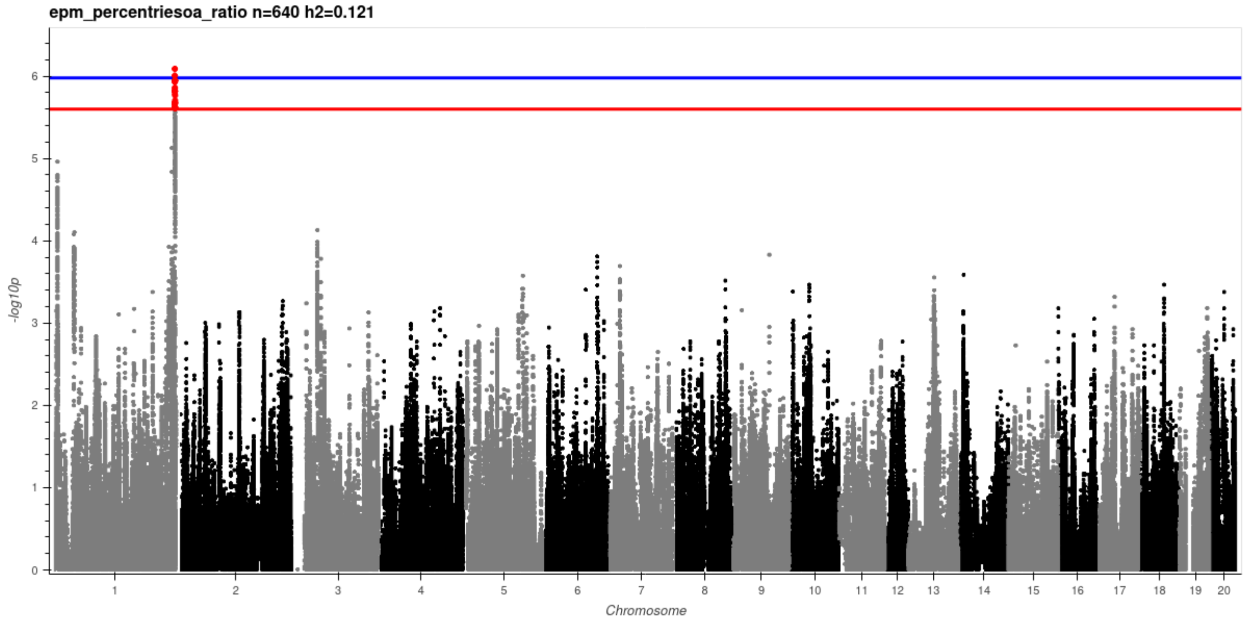

EPM Open Arm Time

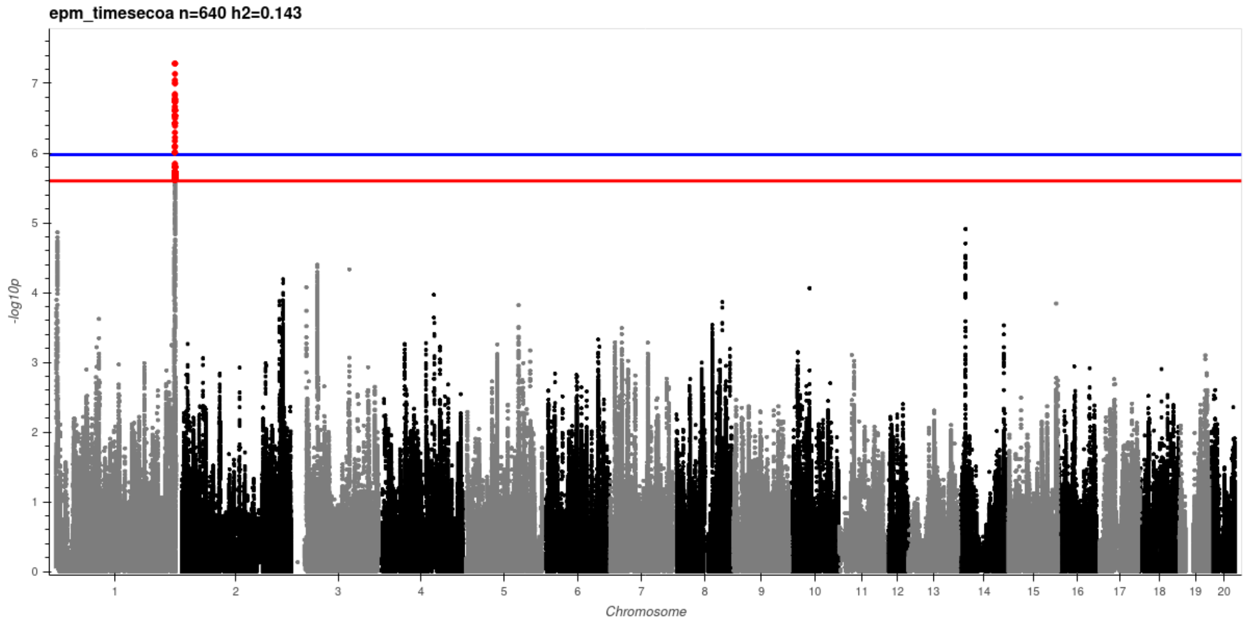

EPM Velocity

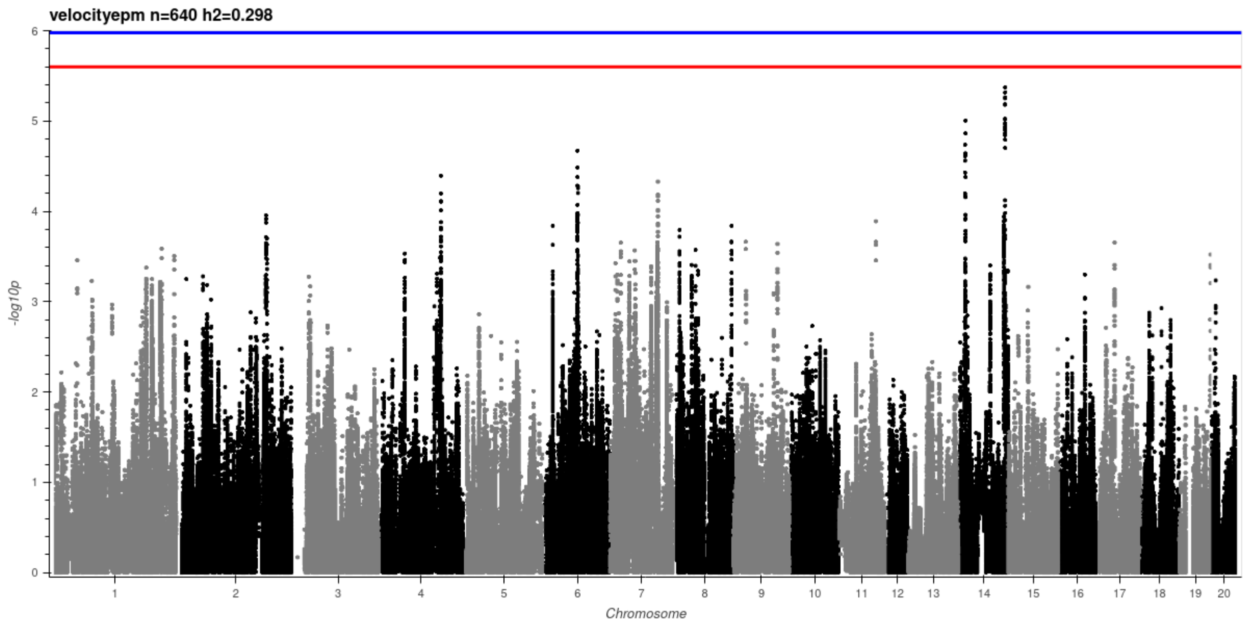

EPM Time Immobile

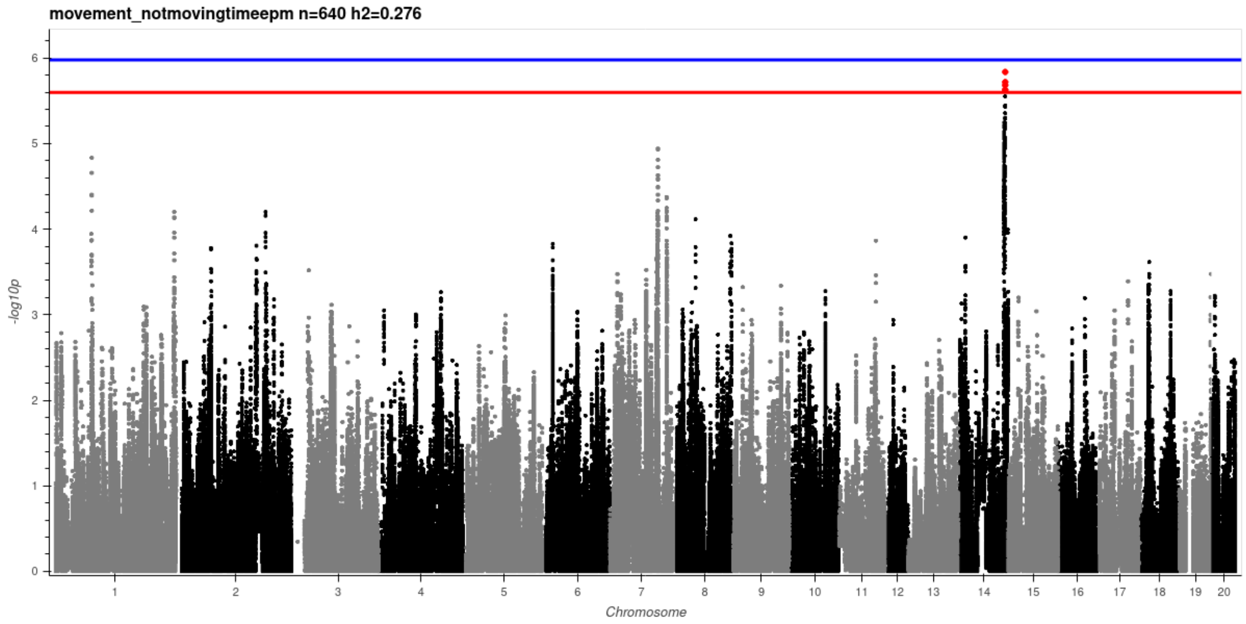

Tail Flick Latency

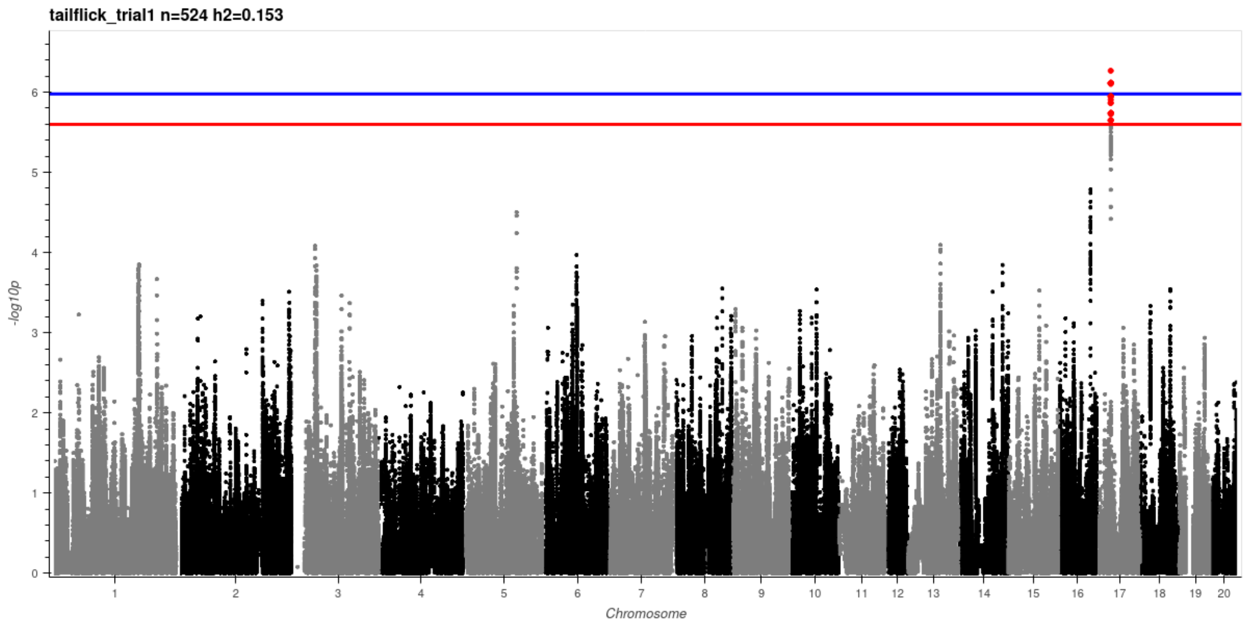

Lateral Locomotor Score

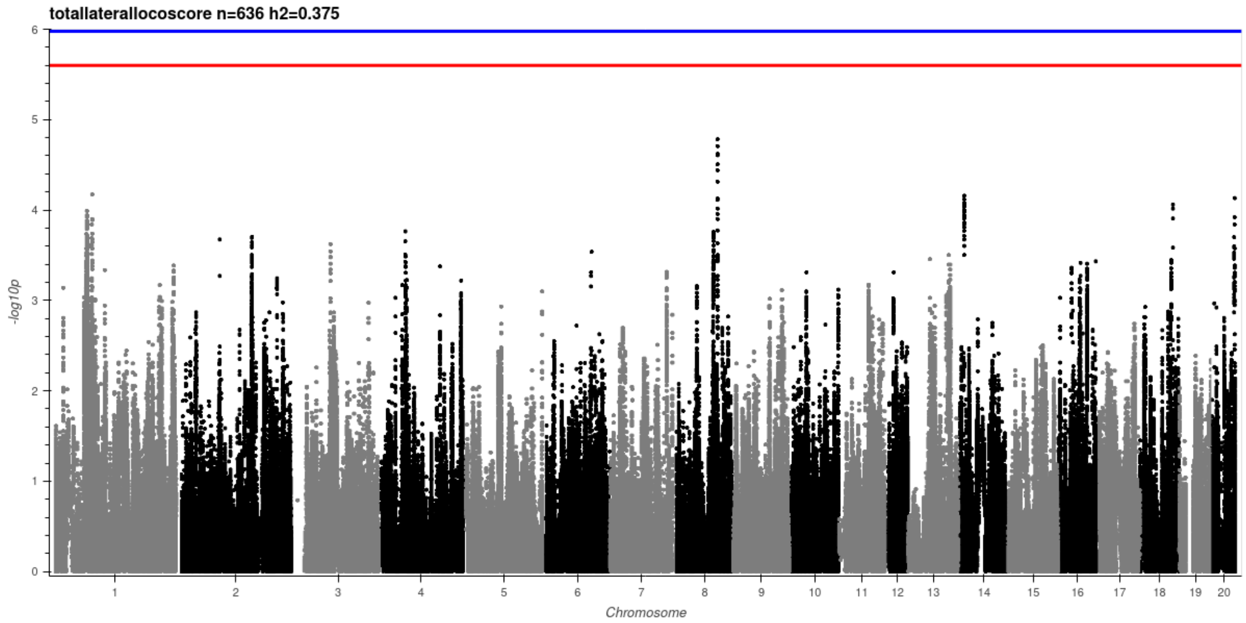

Total Locomotor Score

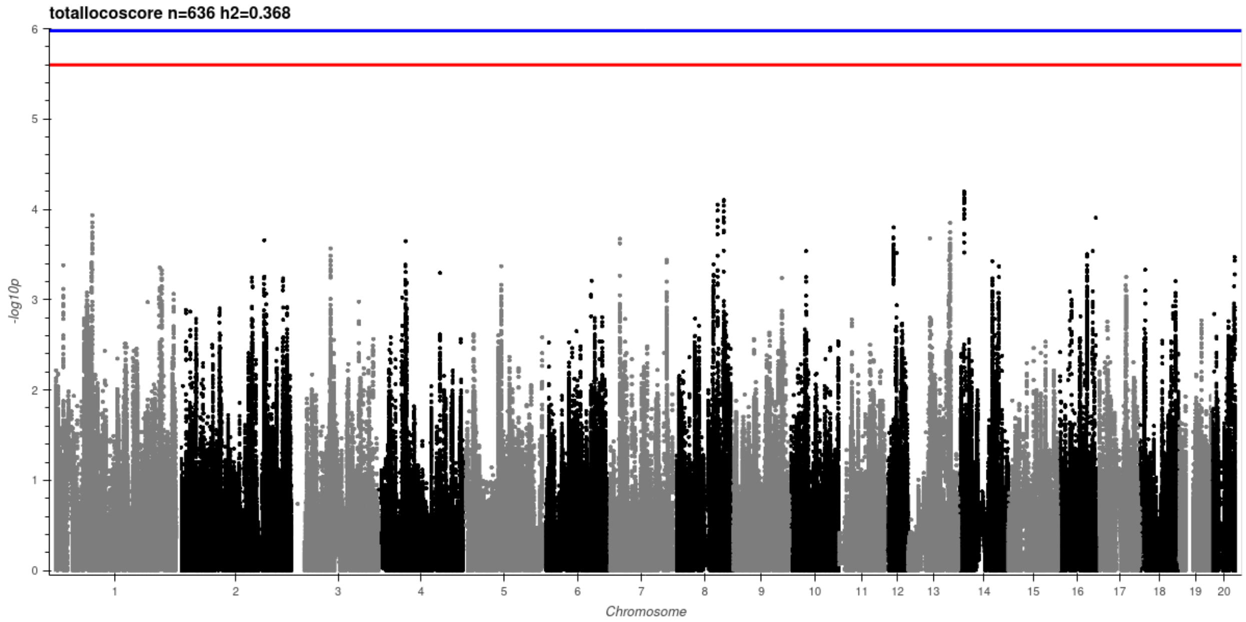

Rearing Locomotor Score

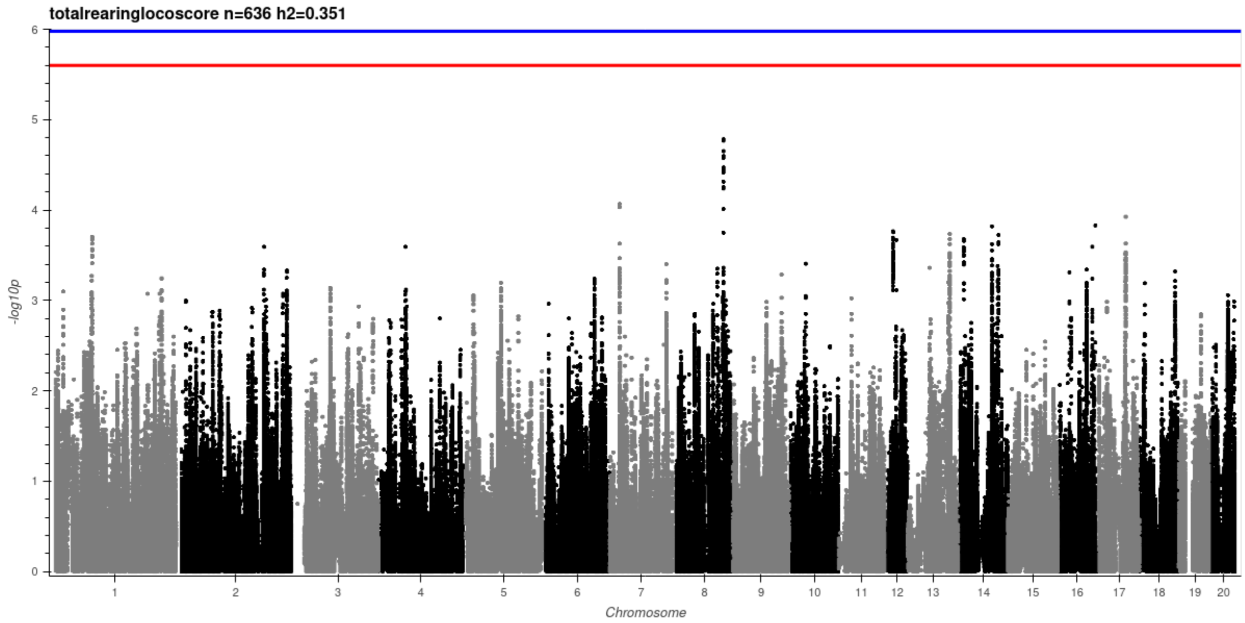

Supplementary Figure S2. Regional association plots for additional significant loci. A) EPM Open Arm Time Ratio at the chromosome 1 locus. B) EPM Open Arm Time at the same chromosome 1 locus. C) EPM Time Immobile at the chromosome 14 locus. Points are colored by LD r² to the lead SNP; axes show genomic position in Mb and −log10P. Horizontal lines mark the genome-wide and suggestive thresholds (−log10P = 5.98 and 5.60, respectively). The gene track displays annotated genes with transcription direction. Panels A and B map to the same chromosome 1 interval as the EPM Percent Time in Open Arms locus in the main figure.

A. EPM Open Arm Time Ratio at the Chromosome 1 Locus

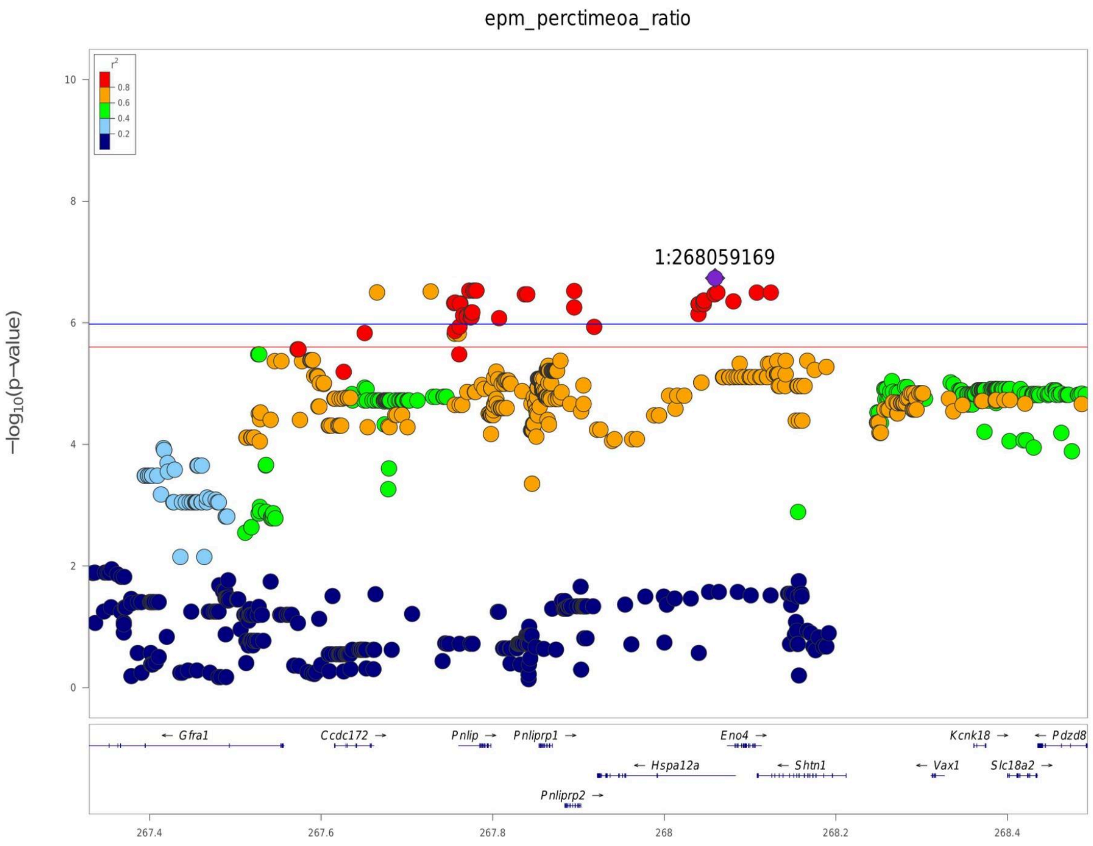

B. EPM Open Arm Time at the Chromosome 1 Locus

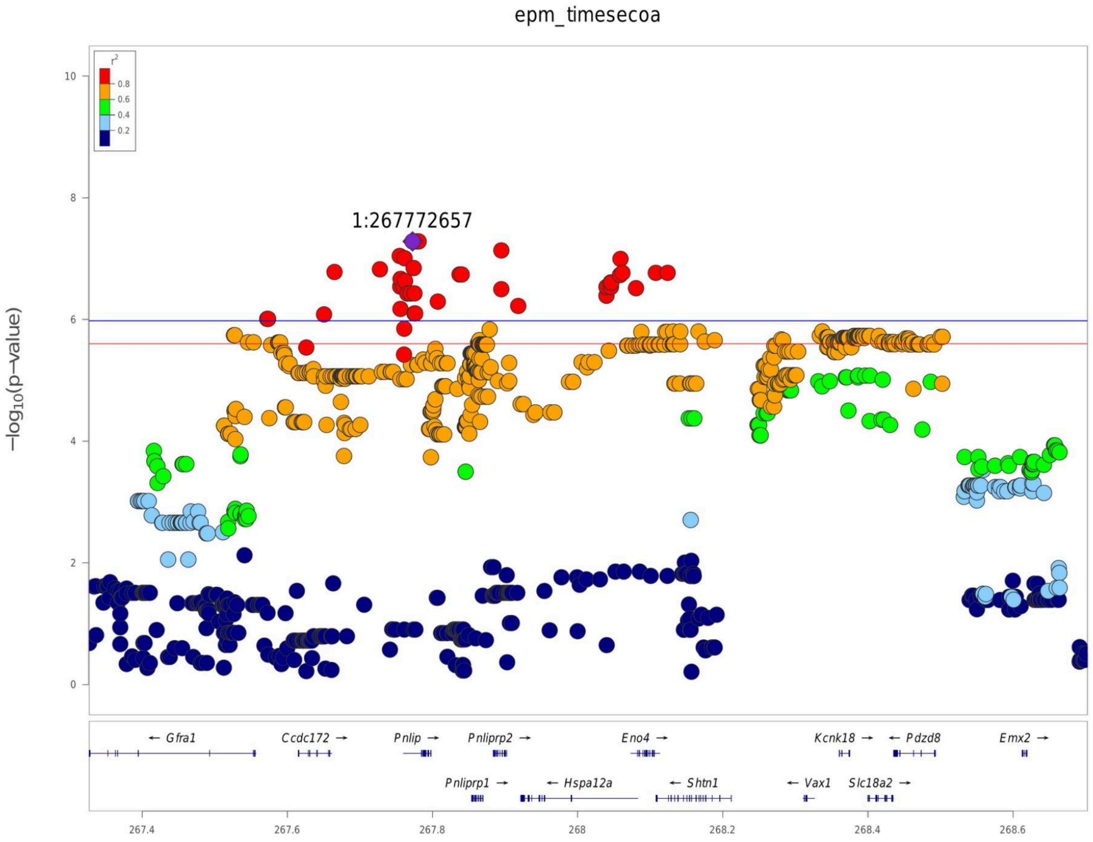

C. EPM Time Immobile at the Chromosome 14 Locus

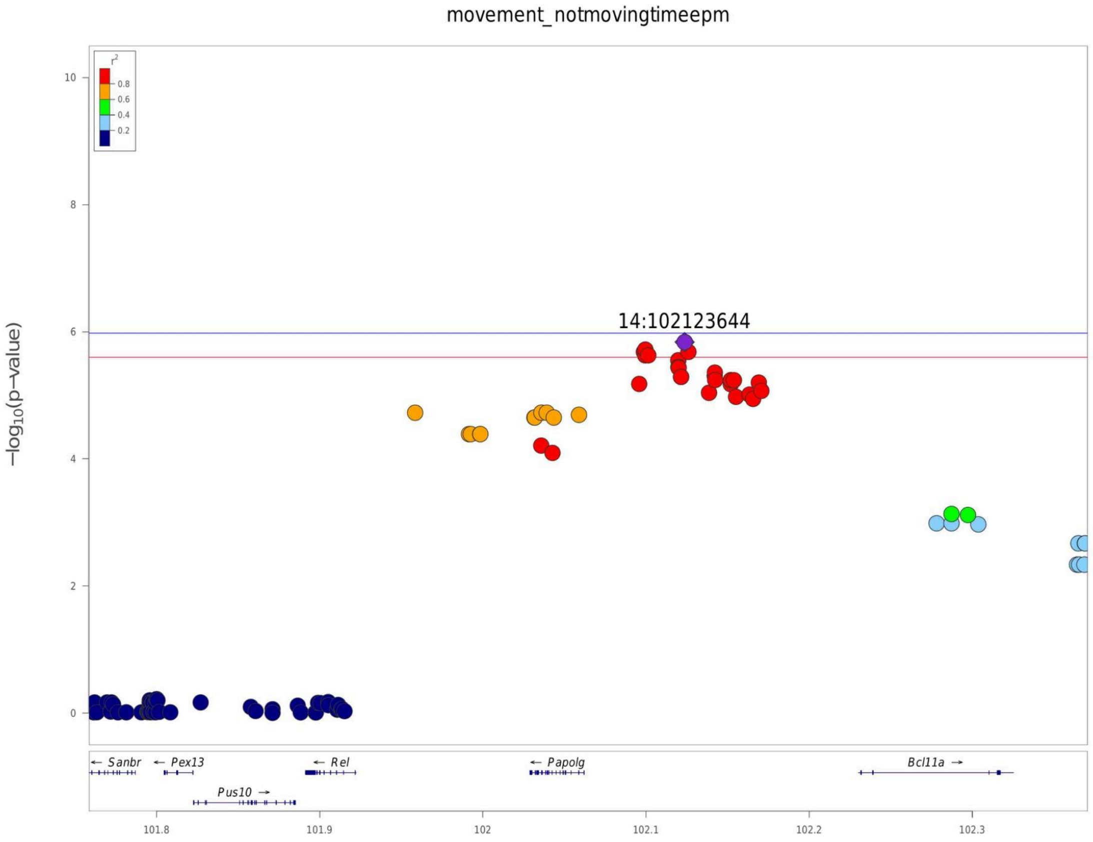

Supplement: Supplement 1 [file media-1.pdf]
